# The novel roles of circRNAs in human cancer

**DOI:** 10.1186/s12943-018-0934-6

**Published:** 2019-01-09

**Authors:** Qingfeng Shang, Zhi Yang, Renbing Jia, Shengfang Ge

**Affiliations:** 1 Department of Ophthalmology, Ninth People’s Hospital, Shanghai Jiao Tong University School of Medicine, No. 12, Lane 833, Zhizaoju Road, Huangpu District, Shanghai, 200001 China; 2 Shanghai Key Laboratory of Orbital Diseases and Ocular Oncology, No. 12, Lane 833, Zhizaoju Road, Huangpu District, Shanghai, 200001 China; 3 CAS Key Laboratory of Tissue Microenvironment and Tumor, Shanghai Institute of Nutrition and Health, Shanghai Institutes for Biological Sciences, University of Chinese Academy of Sciences, Chinese Academy of Sciences, No. 320, Yueyang Road, Xuhui District, Shanghai, 200001 China

**Keywords:** CircRNAs, Cancer, Sponge, Translation, Database

## Abstract

Covalently closed single-stranded circular RNAs (circRNAs) consist of introns or exons and are widely present in eukaryotic cells. CircRNAs generally have low expression levels and relatively stable structures compared with messenger RNAs (mRNAs), most of which are located in the cytoplasm and often act in cell type and tissue-specific manners, indicating that they may serve as novel biomarkers. In recent years, circRNAs have gradually become a hotspot in the field of RNA and cancer research, but the functions of most circRNAs have not yet been discovered. Known circRNAs can affect the biogenesis of cancers in diverse ways, such as functioning as a microRNA (miRNA) sponges, combining with RNA binding proteins (RBPs), working as a transcription factor and translation of proteins. In this review, we summarize the characteristics and types of circRNAs, introduce the biogenesis of circRNAs, discuss the emerging functions and databases on circRNAs and present the current challenges of circRNAs studies.

## Background

In the past few decades, the field of RNA, especially the non-coding RNA(ncRNA)field,has benefitted from the rapid development and application of high-throughput RNA sequencing (RNA-seq) technology [1]. The majority of RNA species in eukaryotic cells is comprised of ncRNA rather than messenger RNA (mRNA), and studies have shown that these ncRNAs play a vital role in physiological and developmental processes. In previous studies, scientists mainly focused on linear ncRNAs, such as long non-coding RNAs (lncRNAs) and microRNAs (miRNAs), indicating that these linear ncRNAs have multiple functions in physiological and pathological processes. As a kind of unique circular ncRNA, circular RNAs (circRNAs) have been previously considered accidental by-products or ‘splicing noise’ with low abundance and little functional potential, resulting from errors during post-transcriptional processing [2]. The first circRNAs were found in the Sendai virus and plant-infected viroids in 1976 by Kolakofsky and Sanger, respectively [3, 4]. Subsequently, only a few circRNAs with or without biological functions were discovered in eukaryotes [5–8]. Now, however, due to the widespread application of new technologies, circRNAs have been recognized and taken seriously in various biological fields. RNA-seq and bioinformatic analysis have proven that thousands of circRNAs are abundant in the brain [9, 10], and recent studies have experimentally confirmed the significant biological functions of circRNAs, especially in the field of cancer [11].

### Characteristics of circRNAs

CircRNAs are covalently closed, single-stranded circular transcripts with no 5′ caps and 3′ poly(A) tails; this structural characteristic makes circRNAs resistant to the digestion of ribonucleases, such as RNase R and exonuclease, and confers a longer half-life than that of linear mRNAs [12]. In addition, most circRNAs are evolutionarily conserved across species [13]. CircRNAs are often expressed at low levels [14–16], implying the possibility that they may act as ‘splicing noise’ with little functional potential. However, multiple circRNAs detected by deep sequencing have been experimentally shown to be expressed more abundantly than their linear counterparts, sometimes even more than 10 times [16, 17]. Most circRNAs are often located in the cytoplasm, consisting of exons, while a small part of circRNAs consisting of introns are located in the nucleus [18], and they are generally expressed in cell type-specific and tissue-specific manners [19].

### Biogenesis of circRNAs

**Fig. 1:**
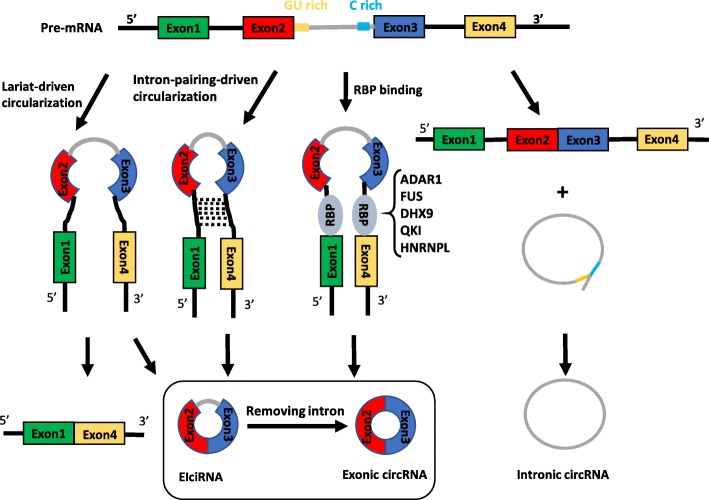
Biogenesis of circRNAs. Different types of circRNAs are generated via different mechanisms. In lariat-driven circularization, the exon skips along with RNA folding, and exonic circRNAs or EIciRNAs are formed with or without removing introns. In intron-pairing-driven circularization, the formation of exonic circRNAs or EIciRNAs are regulated by base pairing in introns on both sides of pre-mRNA. RBPs bind to the specific sequence of introns, which affect the biogenesis of circRNA. In the formation of intronic circRNAs, the intron lariat is formed with the combination of conserved motifs located at upstream and downstream of introns

CircRNAs are produced from precursor mRNA (pre-mRNA), and they are transcribed by RNA polymerase II [20]. The currently discovered circRNAs can be simply sorted into three types according to their different composition and cycling mechanisms: exonic circRNAs, intronic circRNAs and exon-intron circRNAs (EIciRNA). At present, the maturation mechanism of circRNAs is not fully understood. It is inferred that exonic circular RNA is formed by backsplicing [1]. There are currently three hypothetical models explaining the formation of exonic circRNAs: lariat-driven circularization, intron-pairing-driven circularization and RNA binding protein (RBP) mediated circularization [14] (Fig. 1). In the process of forming exonic circRNAs, partial RNA folding occurs during pre-mRNA transcription, and the exon skips along with folding of the RNA. These structural changes result in the formation of specific regions, called lariat structures, in which originally non-adjacent exons become close to each other along with their introns. CircRNA is then formed after the intron sequence is removed by splicing within the lariat structure. This model is defined as lariat-driven circularization. Due to the presence of reverse complement sequences in introns on both sides of pre-mRNA, the complementary pairing of introns on both sides mediates the formation of circRNA. This model is defined as intron-pairing-driven circularization. Additionally, some RNA binding proteins are found to be critical in the formation of circRNAs. The highly conserved RNA-editing enzyme ADAR can bind double-stranded RNAs by targeting double-stranded ALU repeats in human cells [21–23]. ADAR1 antagonizes circRNA biogenesis through A-to-I editing of intron pairs flanking circularized exons, thus diminishing the complementarity and stability of these intron pair interactions [9, 23, 24]. DHX9, an abundant nuclear RNA helicase, has a unique domain organization that resembles ADAR. Silencing DHX9 leads to increased circRNA production through unwinding RNA pairs flanking circularized exons in general. Interestingly, there is a conserved RNA-independent interaction between ADAR (p150) and DHX9 in mouse and human cells, and co-depletion of DHX9 and ADAR can even promote more circRNA production [25]. Errichelli et al. revealed that FUS regulates circRNA formation by binding introns flanking back-splicing junctions in mouse embryonic stem cell (ESC)-derived motor neurons [26]. Heterogeneous nuclear ribonucleoprotein L (HNRNPL) was found to directly regulate the alternative splicing of multiple RNAs. A recent study found that HNRNPL was also involved in the regulation of circRNAs in prostate cancer [27]. Quaking (QKI), as a kind of alternative splicing factor, promotes circRNA generation by binding to its intronic binding motifs. The addition of a synthetic QKI binding motif into introns was sufficient to induce de novo circRNA formation [28]. With respect to intronic circRNAs, it is currently believed that some introns form lariat structures during splicing, but most will be degraded rapidly by debranching, and only some containing the essential nucleic acid sequences, such as a seven nucleotide GU-rich motif near the 5′ splicing site and an eleven nucleotide C-rich motif near the branch point, will not be debranched after splicing, thus forming intronic circRNA [29]. EIciRNAs are composed of exons and introns. In the formation of exonic circRNAs, introns that surround the exons are usually spliced out, however, in some cases, they are retained and are thus named EIciRNA [30].

### Function of circRNAs

#### circRNA can act as ceRNA

The ceRNA(competing endogenous RNA) hypothesis indicates that miRNA can affect mRNA stability and transcription at the post-transcriptional level by binding to target genes; thus, RNA can also affect miRNAs [31]. This hypothesis mainly involves three kinds of RNAs, including mRNA, transcribed pseudogenes and lncRNA [32], and now circRNA, which has followed lncRNA in becoming a new research hotspot among the ceRNA family.

**Fig. 2:**
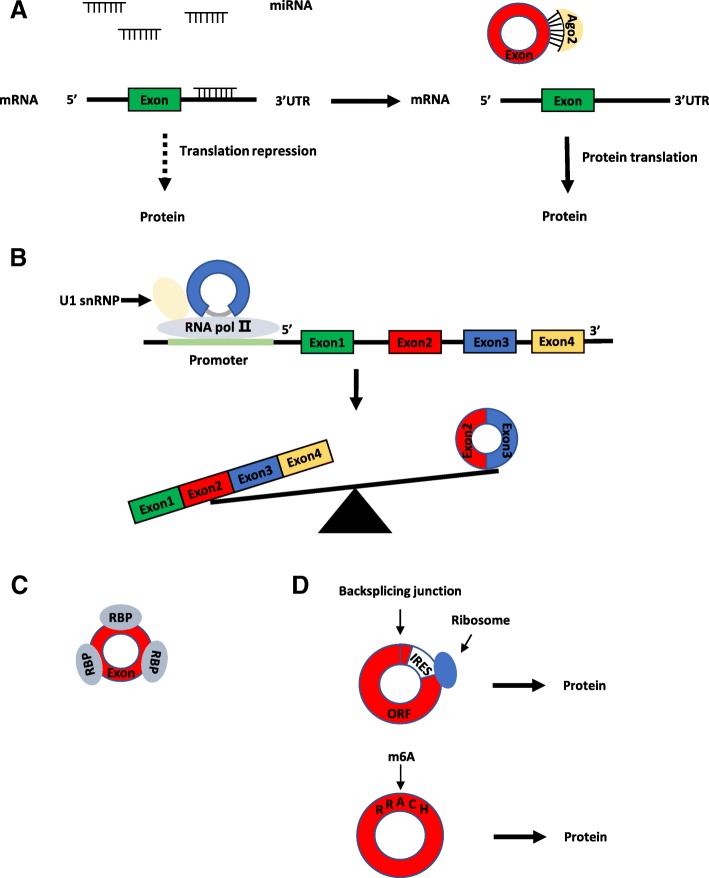
Functions of circRNAs. **a** CircRNAs can sequester miRNAs to regulate the target gene of the miRNAs. **b** CircRNAs can promote parental gene transcription through binding to RNA pol II and U1 snRNP. **c** circRNAs can bind to proteins and work as protein decoys. **d** Some circRNAs can encode proteins driven by IRES or m6a modification

Many circRNAs have different types and amounts of miRNA binding sites that can specifically bind to miRNAs, thereby reducing miRNA activity and upregulating the expression of miRNA-related target genes [33] (Fig. 2a). Researchers found that the circRNA ciRS-7 (circular RNA sponge for miR-7) has more than 60 conserved binding sites for miRNA-7 [34]. In human and mouse brain tissue, ciRS-7 acts as a molecular sponge of miR-7 and inhibits functions of miRNA, which in turn positively regulates miR-7 target genes. Recently, a study found that a long non-coding RNA Cyrano, a circRNA ciRS-7, and two microRNAs miR-671 and miR-7 form a regulatory network through sponge function in the mammalian brain [35]. This study reveals a molecular regulatory network composed entirely of non-coding RNAs and found that there may be unknown new mechanisms regulating the degradation of circRNA. Studies also revealed that circMTO1 was mainly located in the cytoplasm and that its overexpression could decrease cell proliferation and migration levels in vitro or in vivo by targeting miR-9 and increasing p21 expression, which is the target of miR-9 [36]. Several research groups found that a well-known circRNA, circHIPK3, derived from exon 2 of the HIPK3 gene, contains 1099 nucleotides and is abundant in multiple human tissues. In addition, circHIPK3 acts as a sponge for a variety of miRNAs, including the miR-124, miR-558, miR-30 families, miR-7, miR-4288, miR-654, miR-193a, miR-379 and miR-29b [17, 37–47]. Other studies indicated that circRNA ITCH inhibits bladder cancer and glioma progression in the circITCH-miR-17/miR-224-p21/PTEN axis and circITH-miR-214-ITCH axis [48, 49]. In conclusion, these findings mentioned above support the idea that circRNAs acting as miRNA sponges may be a common phenomenon in cancer.

#### CircRNAs act as transcriptional regulators

Although multiple reports support the role of circRNAs as sponges for miRNAs, some scholars have indicated that some intronic circRNAs and EIciRNAs can regulate protein production by regulating gene expression in a transcriptional or post-transcriptional manner [1, 14] (Fig. 2b). EIciRNA, such as EIciPAIP2 and EIciEIF3J, is mainly located in the nucleus and it can interact with the U1 small nuclear ribonucleoprotein(snRNP) and then promote parental gene transcription through binding to RNA polymerase II (RNA pol II) [30]. In addition, one study found that the second exon of the splicing factor MBL can cyclize to form a circRNA, which competes with the linear splicing of pre-mRNA and affects the formation of linear RNA to regulate the expression of related genes [20].

#### CircRNAs bind to proteins

Just as linear RNAs have been revealed to bind to proteins, several studies have shown that certain circRNAs can work as protein decoys, which sequester proteins by harbouring binding sites for specific proteins and block protein activity by working as competing elements (Fig. 2c). For example, circRNA, such as ciRS-7 and SRY, can degrade Argonaute 2 (Ago2) protein and inhibit transcription of mRNA [34, 50]. Circ-Foxo3 was mainly located in the cytoplasm, and it can bind to p21 and CDK2 and induce cell cycle arrest via the formation of the circ-Foxo3-p21-CDK2 ternary complex [51]. In addition, studies have found that circ-Foxo3 can also interact with senescence-related proteins ID1 and E2F1 and tumour-related proteins HIF1α and FAK [52]. Exonic circRNA cia-cGAS (circular RNA antagonist for cGAS) is formed by exons 4, 5 and 6 of D430042O09Rik gene transcripts and is mainly located in the nucleus. Cia-cGAS binds to cGAS protein and inactivates the enzymatic activity of cGAS in long term haematopoietic stem cells (LH-HSCs) [53].

#### CircRNAs can be translated

As non-coding RNAs, circRNAs were once considered to fail to translate via cap-dependent mechanisms due to their lack of a 5’cap structure and a poly (A) tail. Additionally, previous polysome gradient analysis and ribosome profiling showed that most circRNAs are not associated with polysomes [14]. Therefore, circRNAs were generally considered to be non-coding RNAs due to lack of evidence for translation. However, most circRNAs consist of exons are mainly located in the cytoplasm. They may be related to the ribosome to some extent. Recently, strong evidence from many research groups has shown that circRNAs can encode proteins. Some researchers constructed a circular RNA containing an infinite reading frame, demonstrating that this type of circular RNA can mimic DNA rolling circle amplification (RCA) for RNA translation in prokaryotes [54]. Moreover, based on the discovery of the rolling circle translation of circular RNA in prokaryotes, researchers have speculated and verified that this model is equally applicable to the translation system of eukaryotic cells [55]. Based on high-throughput phenotypic screening, Legnini et al. identified circ-ZNF609, a functional circular RNA expressed in murine and human myoblasts that controls myoblast proliferation [56]. Circ-ZNF609 contains an open reading frame that passes the backsplicing junction and is associated with heavy polysomes, and it can be translated into a protein in a splicing-dependent and cap-independent manner. Moreover, Zhang et al. found that the circRNA circFBXW7, formed by exon 3 and exon 4 of the gene FBXW7, can be translated into a novel 21-kDa protein, which was termed FBXW7-185aa [57]. CircFBXW7 and FBXW7-185aa levels are reduced in glioma, and circFBXW7 expression was positively associated with glioblastoma patient overall survival. Moreover, FBXW7-185aa inhibited glioma proliferation and cell cycle acceleration in vitro and in vivo through reducing the half-life of c-Myc by antagonizing USP28-induced c-Myc stabilization. Recently, Zhang et al. revealed that circ-SHPRH containing an open reading frame (ORF) driven by the internal ribosome entry site (IRES) can translate a functional protein, named SHPRH-146aa, in glioma [58]. Interestingly, the translation pattern of circ-SHPRH is quite similar to that of circular RNA of the virusoid associated with rice yellow mottle virus by using overlapping genetic codes to generate a ‘UGA’ stop codon. CircRNA is not easily digested by ribonuclease and is more stable than linear RNA due to its natural structural properties. Therefore, by constructing engineered exogenous circRNA, cyclizing mRNA can effectively solve the problem of the short half-life of linear mRNA, thereby expressing proteins efficiently and permanently in eukaryotic cells [59].

Interestingly, the translation of circRNAs can be mediated not only by IRES but N6-methyladenosine (m6a) can also drive the translation of circular RNA (Fig. 2d). Yang et al. found that approximately 13% of the total circRNA had a m6A modification, that the circRNA carrying the m6A modified motif, the RRACH fragment (R = A or G; H = A, U or C) can translate the polypeptide intracellularly, and that the translation efficiency of the circRNA is affected by its m6A modification level [60].

### Databases of circRNAs

With the development of circRNA field, many databases about circRNAs have been built to facilitates the analysis of circRNAs (Table 1). The Circbase, CIRC pedia v2 and Deepbase 2.0 databases contain numerous circRNAs about different species with detailed information [61–63]. The CircRNADb database contains genomic information, exon splicing, genome sequence, IRES, ORF and references about 32,914 human exonic circRNAs [64]. The Circnet and Starbase v2.0 databases offer researchers the circRNA-miRNA-gene regulatory networks with the application of bioinformatics [65, 66]. The CSCD and CircInteractome databases can be used as tools to predict miRNA response elements(MRE)and RBP [67, 68]. The CirclncRNAnet database provides researchers an easy way to analysis sequencing results [69]. The ExoRBase database provides 58,330 circRNAs existed in human blood exosomes [70]. The circRNADisease database records experimentally verified circRNAs in mutiple diseases [71].

**Table 1:** Database about circRNAs

Database	Website	Function	Reference
Circbase	http://www.circbase.org/	CircBase merged and unified data sets of circRNAs and the evidence supporting their expression, and also provided scripts to identify known and novel circRNAs in sequencing data	[61]
CIRCpedia v2	http://www.picb.ac.cn/rnomics/circpedia	CIRCpedia contained comprehensive circRNA annotation from over 180 RNA-seq datasets across six different species	[62]
DeepBase v2.0	http://rna.sysu.edu.cn/deepBase/	DeepBase v2.0 annotated 14,867 human circRNAs	[63]
CircRNADb	http://202.195.183.4:8000/circrnadb/circRNADb.php	CircRNADb provided the detailed information of the circRNA, including genomic information, exon splicing, genome sequence, IRES, ORF and references	[64]
Circnet	http://circnet.mbc.nctu.edu.tw/	Circnet generated an integrated regulatory network that illustrated the regulation between circRNAs, miRNAs and genes	[65]
Starbase v2.0	http://starbase.sysu.edu.cn/	StarBase v2.0 systematically identified the RNA-RNA and protein-RNA interaction networks from 108 CLIP-Seq data sets generated by 37 independent studies	[66]
CSCD	http://gb.whu.edu.cn/CSCD/	CSCD predicted the microRNA response element sites, RNA binding protein sites and potential open reading frames for each circRNA	[67]
CircInteractome	http://circinteractome.nia.nih.gov/	CircInteractome searched public circRNA, miRNA, and RBP databases to provide bioinformatic analyses of binding sites on circRNAs and additionally analyzes miRNA and RBP sites on junction and junction-flanking sequences	[68]
CirclncRNAnet	http://app.cgu.edu.tw/circlnc/	CirclncRNAnet provided a “one-stop” resource for in-depth analyses of ncRNA biology	[69]
ExoRBase	http://www.exorbase.org/	ExoRBase provided annotation, expression level and possible original tissues about 58,330 circRNAs in human blood exosomes	[70]
CircRNADisease	http://cgga.org.cn:9091/circRNADisease/	CircRNA Disease provided experimentally supported circRNA and disease associations	[71]

### CircRNAs in cancer

To date, there has been multiple functional cancer-associated circRNAs screened and identified by different research groups (Table 2). These circRNAs play as oncogenes or tumor suppressors in multiple cancers and affect cancer phenotype via diverse ways.

**Table2:** A list of circRNAs in tumorigenesis

Cancer types	CircRNA (Circbase ID)	Roles in cancer	Cancer phenotype	Sponge miRNA	Target gene	Clinical significance	Reference
Hepatocellular carcinoma	circMTO1 (hsa_circ_0007874)	Tumor suppressor	Inhibits cell proliferation and invasion and promotes apoptosis	miR-9	p21	Overall survival	[36]
	cSMARCA5 (hsa_circ_0001445)	Tumor suppressor	Inhibits cell growth and migration	miR-17-3p and miR-181b-5p	TIMP3	Aggressive characteristics; overall survival, recurrence-free survival after hepatectomy	[72]
	circZKSCAN1 (hsa_circ_0001727)	Tumor suppressor	Inhibits cell growth, migration and invasion	/	/	Tumor numbers, vascular invasion, tumor grade, cirhosis	[73]
Breast cancer	circ-Ccnb1 (hsa_circ_0072758)	Tumor suppressor	Decreases cell proliferation and promote apoptosis	/	/	/	[74]
	circGFRA1 (hsa_circ_0005239)	Oncogene	Promotes cell proliferation and inhibits apoptosis	miR-34a	GFRA1	Clinical outcomes	[75]
	circEPSTI1 (hsa_circ_0000479)	Oncogene	Promotes cell proliferation and inhibits apoptosis	miR-4753 and miR-6809	BCL11A	Overall survival	[76]
Leukaemia	f-circRNA	Oncogene	Promotes cellular transformation, cell viability and resistance upon therapy	/	/	/	[11]
Lung cancer	circ0006916 (hsa_circ_0006916)	Tumor suppressor	Inhibits cell proliferation	miR-522-3P	PHLPP1	/	[77]
	F-circEA	Oncogene	Promote cell migration and invasion	/	/	Novel biomarker for diagnosis	[78]
Glioma	circ-FBXW7	Tumor suppressor	Inhibited cell proliferationand cell cycle acceleration	/	/	Overall survival	[57]
	circ-SHPRH (hsa_circ_0001649)	Tumor suppressor	Reduced malignant behavior and tumorigenicity	/	/	/	[58]
Colorectal cancer	circCCDC66 (hsa_circ_0001313)	Oncogene	Promoted cell proliferation, migration and invasion	miR-33b and miR-93	MYC	Biomarker for cancer detection and prognosis	[79]
	circHIPK3 (hsa_circ_0000284)	Oncogene	Promoted cell proliferation, migration, invasion, and inhibited apoptosis	miR-7	FAK,IGF1R, EGFR, YY1	Metastasis, advanced clinical stage; overall survival	[39]
	circITGA7 (hsa_circ_0026782)	Tumor suppressor	Suppressed cell proliferation and metastasis	miR-370-3p	NF1	Tumor size, lymph metastasis, distant metastasis; TNM stage	[80]
Bladder cancer	circHIPK3 (hsa_circ_0000284)	Tumor suppressor	Inhibited cell migration and invasion	miR-558	HPSE	/	[38]
	circ-ITCH	Tumor suppressor	Inhibited cell growth, colony formation, migration and invasion	miR-17 and miR-224	p21, PTEN	/	[49]

#### CircRNA in hepatocellular carcinoma

Several studies have found that multiple circRNAs play a tumour suppressor role in hepatocellular carcinoma. CircMTO1 is underexpressed in hepatocellular carcinoma and affects the expression of downstream P21 protein by targeting miR-9 [36]. It was also revealed that circMTO1 is associated with a prognosis of hepatocellular carcinoma, indicating that circMTO1 may be a prognostic biomarker for hepatocellular carcinoma. Yu et al. found that the circRNA cSMARCA5 is downregulated in hepatocellular carcinoma and inhibits the proliferation and metastasis of hepatocellular carcinoma by sponging miR-181b-5p and miR-17-3p, thereby regulating TIMP3 expression [72]. In addition, the researchers also explored upstream of cSMARCA5 and found that the expression level of RNA helicase DHX9 was upregulated in hepatocellular carcinoma and that cSMARCA5 was negatively regulated. In addition, the study found that ZKSCAN1 mRNA and circular RNA circZKSCAN1 can both regulate the proliferation, invasion and migration of hepatocellular carcinoma through different pathways [73].

#### CircRNA in breast cancer

Derived from exon 4 and exon 5 of the CCNB1 gene, the circRNA circ-Ccnb1 was downregulated in breast cancer and mainly localized in the nucleus in breast cancer cells [74]. Based on an RNA pull-down assay to screen interacting proteins, it was found that circ-Ccnb1 can interact with p53 in p53 wild-type cells via H2AX but instead interacts with Bclaf1 in p53 mutant cells via H2AX, resulting in the induction of cell death in breast cancer. In triple-negative breast cancer, circGFRA1 acts as miR-34a ceRNA to regulate GFRA1 expression [75], while circEPSTI1 promotes triple-negative breast cancer cell proliferation through a novel pathway by sponging miR-4753 and miR-6809 and upregulating the oncogene BCL11A [76].

#### CircRNA in leukaemia

Multiple types of tumour cell genomes have chromosomal translocations and rearrangements, resulting in the formation of irrelevant gene recombination and fusion mRNAs that are ultimately translated into fusion proteins. Studies found that in acute promyelocytic leukaemia cells, the PML-RARa fusion gene can not only translate the fusion protein but also form a fusion circRNA f-circM9 [11]. A gain of function assay and loss of function assay have shown that f-circM9 plays a proto-oncogene role that contributes to cellular transformation in acute promyelocytic leukaemia. Additionally, f-circRNA conferred resistance to therapy in vivo and synergizes with other carcinogenic factors such as fusion proteins to promote cancer development.

#### CircRNA in lung cancer

Dai et al. found that circRNA circ0006916 acts as a tumour suppressor in lung cancer by binding to miR-522-3p and inhibiting PHLPP1 activity, thus inhibiting cell proliferation via slowing down the cell cycle process rather than by promoting apoptosis [77]. In addition, the RNA-binding protein TNRC6A binds to the flanking intron sequence of circ0006916 and regulates circRNA expression. This study successfully revealed the upstream mechanisms of circRNA circ0006916, and clearly validated the mechanism of circRNA in cell malignant transformation. Fusion genes not only exist in blood diseases but also in solid tumours. A novel circRNA, named as F-circEA, was generated from the EML4-ALK fusion gene by back-splicing in non-small cell lung cancer (NSCLC) [78]. F-circEA, independent of the EML4-ALK linear transcript and fusion protein, contributes to cancer progression by promoting cell migration and invasion. Notably, the evidence that F-circEA mainly exists in the plasma of EML4-ALK-positive NSCLC patients suggests that F-circEA could be a potentially novel biomarker for the diagnosis of EML4-ALK-positive NSCLC patients.

#### CircRNA in glioma

Yang et al. revealed that the endogenous circRNA circFBXW7, comprised of exon 3 and exon 4 of the FBXW7 gene, could encode a novel 21-kDa protein, named FBXW7-185aa, driven by the IRES [57]. This protein can synergize with the protein encoded by FBXW7 to antagonize the stability of the proto-oncogene c-Myc and inhibit the progression of glioma. A study revealed the presence of the downregulated circRNA circ-SHPRH in gliomas, which translates a new 17 kDa protein that named SHPRH-146aa by using overlapping genetic codes [58]. SHPRH-146aa protects full-length SHPRH from degradation by the ubiquitin proteasome. Stabilized SHPRH sequentially ubiquitinates proliferating cell nuclear antigen (PCNA) as an E3 ligase, leading to inhibited cell proliferation and tumourigenicity.

#### CircRNA in colorectal cancer

Recently, a study found that the circular RNA circCCDC66 is upregulated in a variety of tumour cells and is associated with poor prognosis of colorectal cancer [79]. CircCCDC66 regulates MYC expression by binding to miR-33b, miR-93, and miR-185. The upregulated circRNA circHIPK3 can promote proliferation, migration, invasion, and inhibit apoptosis in vitro and promote colorectal cancer growth and metastasis in vivo by increasing the expression levels of miR-7 targeting proto-oncogenes, such as FAK, IGF1R, EGFR, YY1 [39]. Researchers indicated a tumour suppressor role for circITGA7 and ITGA7 in colorectal cancer and revealed that circITGA7 inhibits proliferation and metastasis of colorectal cancer cells by suppressing the Ras signalling pathway and promoting the transcription of ITGA7 [80].

#### CircRNA in bladder cancer

Studies from Li et al. revealed that circRNA circHIPK3 was significantly downregulated in bladder cancer tissues and cell lines and was negatively correlated with bladder cancer grade, invasion as well as lymph node metastasis, respectively [38]. CircHIPK3 contains two critical binding sites for miR-558 and can suppress HPSE expression to inhibit migration, invasion and angiogenesis of bladder cancer cells. Yang et al. reported that circular RNA circ-ITCH regulates p21 and PTEN expression by sponging miR-17/miR-224, thus inducing cell cycle arrest and apoptosis [49].

### Challenges

With the development of sequencing technology and bioinformatics, an increasing number of circRNAs are gradually being discovered and have become hotspots in the field of RNA. However, there are still quite a few challenges that need to be addressed. First, there is no uniform naming convention for circRNAs at present, which means researchers in different fields use different names when studying the same circRNA molecules, thus causing confusion. For example, cirs-7 indicates that this circular RNA acts as a sponge for miR-7, and CDR1as refers to a circRNA that is related to the CDR1 gene, but both actually refer to the same circRNA. Second, the current databases of circRNA are still not perfect. Although researchers can predict the function of circRNA by using multiple databases, there is no circRNA database related to tumour prognosis, which makes it difficult to screen functional circRNA after RNA-seq. In addition, although sponging miRNAs is the most classical functional model of circRNA, whether this effect between miRNA and circRNA is universal is questioned. It has been reported that, based on the results of bioinformatics and experimental verification, the circRNA-miRNA functional model has a negative result [81]. Most of the circRNAs currently studied have fewer binding sites for miRNAs, compared with the relationship between CDR1as and miR-7, which greatly reduces the possibility of circRNAs binding to miRNAs. Therefore, the sponge functional model of circRNA and miRNA needs further research. Also, compared with other members of the RNA family, the mechanisms and functional models of the currently reported circRNAs are not diverse, which needs to be further explored, and there are problems for beginners in that the experimental techniques are relatively difficult.

## Conclusion

The discovery of circRNA enriches cognition of biological evolution, increases understanding of RNA, and makes it a hotspot in the field of cancer research, which has given researchers a new direction in which to investigate the occurrence and development of tumours. Although knowledge of the precise mechanism of the formation, transportation and function of circRNAs in tumours is still very limited, with the application of new technologies and the constant efforts of scientists, the mystery of circRNA will eventually be solved.

## Abbreviations

ADAR: Adenosine deaminase
Ago2: Argonaute 2
BCL11a: B cell CLL/lymphoma 11A
Bclaf1: BCL2 associated transcription factor 1
CCNB1: Cyclin B1
ceRNA: Competing endogenous RNA
cia-cGAS: Circular RNA antagonist for cGAS
circRNAs: Circular RNAs
ciRS-7: Circular RNA sponge for miR-7
DHX9: DExH-box helicase 9
E2F1: E2F transcription factor 1
EGFR: Epidermal growth factor receptor
EIciRNA: Exon-intron circRNAs
ESC: Embryonic stem cell
FAK: Protein tyrosine kinase 2
FUS: FUS RNA binding protein
GFRA1: GDNF family receptor alpha 1
H2AX: H2A histone family member X
HIF1α: hypoxia inducible factor 1 subunit alpha
HNRNPL: Heterogeneous nuclear ribonucleoprotein L
HPSE: Heparanase
ID1: Inhibitor of DNA binding 1, HLH protein
IGF1R: Insulin like growth factor 1 receptor
IRES: Internal ribosome entry site
ITCH: Itchy E3 ubiquitin protein ligase
ITGA1: Integrin subunit alpha 1
LH-HSCs: Long term haematopoietic stem cells
lncRNA: Long non-coding RNAs
m6a: N6-methyladenosine
MBL: Muscleblind
miRNA: MicroRNA
MRE: MiRNA response elements
mRNAs: Messenger RNAs
ncRNA: Non-coding RNA
NSCLC: Non-small cell lung cancer
ORF: Open reading frame
p21: Cyclin dependent kinase inhibitor 1A
p53: Tumor protein p53
PCNA: Proliferating cell nuclear antigen
pre-mRNA: Precursor mRNA
PTEN: Phosphatase and tensin homolog
QKI: Quaking
RBPs: RNA binding proteins
RCA: Rolling circle amplification
RNA pol II: RNA polymerase II
RNA-seq: RNA sequencing
RRACH: m6A modified motif
snRNP: small nuclear ribonucleoprotein
SRY: Sex determining region Y
TIMP3: TIMP metallopeptidase inhibitor 3
YY1: YY1 transcription factor
ZKSCAN1: Zinc finger with KRAB and SCAN domains 1
